# Safety and Early Results for Off-Label Use of Intranasal Calcitonin for Treatment of Nondisplaced Acromial and Scapular Spine Stress Fractures After Reverse Total Shoulder Arthroplasty

**DOI:** 10.5435/JAAOSGlobal-D-24-00045

**Published:** 2024-03-28

**Authors:** Krishna Mandalia, Lawrence Gulotta, Glen Ross, Sarav Shah

**Affiliations:** From the Tufts University School of Medicine, Boston, MA (Mr. Mandalia); the Department of Orthopedic Surgery, New England Baptist Hospital, Boston, MA (Mr. Mandalia and Dr. Shah); New England Shoulder and Elbow Center, Boston, MA (Mr. Mandalia, Dr. Ross, and Dr. Shah); and the Sports Medicine Institute, Hospital for Special Surgery, Manhattan, NY (Dr. Gulotta).

## Abstract

Immobilization for acromial and scapular spine stress AU4fractures (AF/SSF) after reverse total shoulder arthroplasty (RSA) is associated with patient dissatisfaction. Our study reports the effects and safety of intranasal calcitonin alongside sling immobilization on pain and function in the treatment of AF/SSF after RSA. The treatment was regimented calcitonin (salmon) 200 unit/actuation nasal spray (1 spray/day) for 6 weeks with sling immobilization for 4 weeks. Each patient was monitored through blood work. Visual analog scale, American Shoulder and Elbow Surgeons score, and active range of motion were collected preoperatively, postoperatively, at presentation of AF/SSF, and after completion of calcitonin treatment. Two hundred eighty-two RSAs were performed by two board-certified orthopaedic surgeons, of which 18 patients sustained AF/SSF (6.4%). Ten patients met inclusion criteria (nine AFs and one SSF). After calcitonin treatment, patients demonstrated an average improvement of visual analog scale of 5.8 points, active range of motion of 46_, and American Shoulder and Elbow Surgeons score of 43.6 points at average 7.53 months after RSA. No medical complications were reported at 6-month follow-up after calcitonin treatment. The use of intranasal calcitonin was not associated withadverse events including no aberrations/signs of cancer at 6-month follow-up after administration. Calcitonin with sling immobilization markedly improved clinical and functional outcomes of patients with nondisplaced AF/SSF and may be considered by orthopaedic surgeons for symptom management.

Since the inception of reverse total shoulder arthroplasty (RSA), an improved understanding of RSA biomechanics and implant design has facilitated the expansion of RSA indications, which now include displaced proximal humeral fractures in the elderly, inflammatory arthritis, irreparable rotator cuff tears without arthropathy, osteoarthritis with intact rotator cuff, chronic dislocations, and failed total shoulder arthroplasty.^1,2^ As the indications for RSA have expanded, the use of RSA has grown profoundly, and it has become the predominant shoulder arthroplasty performed in the United States since 2016.^3^

The most frequently reported complications of RSA are scapular notching, implant loosening, glenohumeral joint instability, and acromial and scapular spine stress fractures (AF/SSF).^3,4^ As implant designs, techniques, and overall understanding of RSA biomechanics have improved with time, rates of many early complications, such as implant loosening and instability, have markedly decreased in recent years. Despite these advancements, the rate of AF/SSF remains steady at ∼2% to 3%,^3^ with some studies reporting rates as high as 7%.^5^ The prevalence of AF/SSF in patients after RSA has been attributed to the altered biomechanics of the glenohumeral joint and surrounding structures, with increased tension on the deltoid muscle.^3,5,6,7,8,9,10,11^ Among the biomechanical advantages offered by the RSA is the increased deltoid abductor moment arm length, which is because of medialization of the glenohumeral joint center of rotation relative to the native shoulder.^6^ This increase in the deltoid abductor moment arm facilitates a greater capacity to generate torque and ultimately improves deltoid efficiency in supporting external loads and increased joint stability.^6^ While these biomechanical advantages allow the deltoid to provide active range of motion (AROM) in a rotator cuff–deficient shoulder, medialization of the glenohumeral joint center of rotation often involves inferior translation of the humerus and concomitant distalization of the deltoid insertion. Subsequently, this elongates the deltoid beyond its native length, imparting increased physiologic stress on the acromion and scapular spine, and likely contributes to the presence of AF/SSFs observed after RSA.^11-13^

AF/SSFs result in markedly worse pain and functional outcomes after RSA.^9,14-16^ Furthermore, a large area of deltoid origin is involved with AF/SSFs, heightening the concern for markedly altered deltoid function with fracture nonunion or malunion.^15^ Nevertheless, there remains no consensus on how best to treat AF/SSFs. Surgical management of AF/SSFs is associated with markedly higher rates of bony union than nonsurgical approaches; however, this is not accompanied by notable improvement in clinical outcomes when compared with standard conservative approaches.^3,16-18^ In conjunction, surgical management of AF/SSFs is thought to have a high rate of failure.^5^ The currently accepted practices for noninvasive management of AF/SSF involves cessation of physical therapy and immobilization.^5^ However, it has been previously demonstrated that cessation of physical therapy and immobilization alone are associated with high rates of patient dissatisfaction and worse clinical outcomes, as compared with control subjects.^5^ Specifically, Boltuch et al demonstrated that, in comparison with control subjects, the nonsurgical management of AF/SSFs was significantly associated with worse American Shoulder and Elbow Surgeons (ASES), visual analog scale (VAS) pain and function, simple shoulder test, and single assessment numeric evaluation scores as well as worse AROM and with radiographic findings such as scapular rotation and osteolysis.^5,19^ Newer treatment modalities have arisen to help curb the poor functional and clinical outcomes associated with AF/SSFs.

One such treatment is the addition of off-label use of intranasal calcitonin to the current nonsurgical treatment algorithm. Historically, the use of calcitonin has been successful in decreasing pain, increasing bone mineral density (BMD) in osteoporosis, and improving functional outcomes in osteoporotic fractures.^20^ One randomized, double-blind, controlled trial demonstrated that those treated with intranasal calcitonin after internal fixation of hip fractures had markedly greater radiographic fracture fusion compared with placebo, suggesting that intranasal calcitonin may serve a substantial role in fracture healing.^19,21,22^ However, the use of intranasal calcitonin has been of great scrutiny in recent years given a controversial citing of its association with a diagnosis of cancer, leading to its suspension in Europe in 2012.^23^

Thus, the purpose of this study was to report the early results of intranasal calcitonin treatment on pain and functional outcomes for nondisplaced AF/SSF after RSA. We hypothesized that intranasal calcitonin treatment would reduce pain and increase functional status, as determined by VAS pain scores, ASES scores, and AROM, in patients with AF/SSF. While this study did not seek to characterize the entire safety profile of intranasal calcitonin, standard blood work and hepatic function testing were followed for report of any adverse events, including clinical signs of cancer (ie, anemia, serum electrolyte abnormalities, transaminitis).

## Methods

An institutional review board–approved retrospective chart review of all patients in the New England Shoulder and Elbow Center electronic health record system was conducted to identify consecutive patients who were treated for shoulder pathologies by two board-certified orthopaedic surgeons (S.S. and G.R.) from January 2020 to December 2022. Of note, this was a case series of completed treatment of nondisplaced AF/SSF based on surgeon preference. Included patients are those who elected for calcitonin treatment and were initiated on intranasal calcitonin therapy at presentation of fracture. Although studies have shown that patients with a diagnosis of osteoporosis have a higher likelihood of AF, there are no data to suggest that dual-energy X-ray absorptiometry scan after sustaining an AF would affect treatment modalities because no correlation has been demonstrated between conventional BMD (lumbar spine and femur) and each proximal humerus BMD.^24,25^ Thus, dual-energy X-ray absorptiometry scans were not obtained before starting calcitonin treatment.

### Inclusion and Exclusion Criteria

The primary inclusion criteria were patients diagnosed by a combination of clinical examination and imaging with AF/SSF after RSA, who were subsequently treated with an intranasal calcitonin spray. The prescription for intranasal calcitonin spray (200 units/actuation) was intended for use as one spray every day by the nasal route for 42 days, switching between the nostrils sprayed each day. Intranasal calcitonin was used in conjunction to 4 weeks of joint immobilization from an arm sling. Exclusion criteria included the prophylactic prescription of calcitonin (ie, for osteoporosis) and treatment with calcitonin for a diagnosis other than AF/SSF after RSA.

### Primary and Secondary Outcomes

The primary end points of this study were patient-reported outcome measures (PROMs), including the VAS and ASES scores. Secondarily, AROM in the forward flexion plane was reported. Each end point was collected preoperatively, postoperatively, at presentation of AF/SSF, and after the completion of calcitonin treatment. Additional patient data included patient age, sex, time to complication, primary indication for shoulder surgery, and fracture type. CT, bone scan, and/or radiograph data were collected for each patient at presentation of AF/SSF. These images were then evaluated by a board-certified orthopaedic surgeon (S.S.) to further characterize the fractures according their location and fracture union status. Fractures were further classified using the Levy postoperative acromial fracture classification system.^9^ Complete blood count and comprehensive metabolic panel in conjunction to clinical examination were conducted at 6-month follow-up after calcitonin treatment to evaluate for any laboratory abnormalities or clinical signs that may suggest cancer.

### Statistical Analysis

Data were collected using a custom data extraction worksheet. Subsequent statistical and descriptive analyses were conducted on Microsoft Excel. One-sample/paired *t*-tests were conducted to evaluate for statistically significant differences between PROMs at each time point along. Unpaired *t*-tests were conducted to evaluate for statistical differences in PROMs at the post-calcitonin time point (at the conclusion of the 42-day treatment) between patients with Levy type I fractures and those with Levy type II/III fractures. We chose to compare PROMs between those with Levy type I and type II/III fractures given that previous literature has shown that fractures medial to the glenoid face (Levy II/III) have been associated with worse clinical outcomes than those with fractures lateral to the glenoid face (Levy I) during nonsurgical management.^5^ A *P*-value of less than 0.05 was considered statistically significant.

## Results

From January 2020 to December 2022, 282 RSAs were performed, of which 18 patients were diagnosed with an AF/SSF after RSA (6.4%). Of the 18 patients identified, eight were excluded from this study for the following reasons: Four were treated with calcitonin prophylactically before clinical diagnosis, two were treated for postoperative humeral fractures, one experienced septic complications, and one patient was lost to follow-up.

### Patient Demographics and Injury Characteristics

Ten patients in total were included, of which five were male (50%) and five were female (50%). The average age at the time of surgery was 71.3 years, with a range of 52 to 85 years. The primary indications for surgery comprised eight patients with rotator cuff arthropathy, one patient with end-stage arthrosis, and one patient with a proximal humeral fracture. All 10 patients were treated with RSA. The average time to AF/SSF complication was 6.03 months, with a range of 1.3 to 14.4 months. Thus, PROMs were measured after calcitonin treatment regimen completion (an average of 7.53 months from the index RSA procedure). Nine patients presented with an AF, and one patient presented with a SSF. Patient demographics, procedure, and complication characteristics are summarized in Table 1. All patients were followed with hepatic function testing and standard blood work. No patients had medical complications or reported adverse events from intranasal calcitonin treatment, including no signs or diagnoses of cancer at 6-month follow-up.

**Table 1:** Patient Demographics, Procedure, and Complication Characteristics

Patient	Age (y)	Sex	Primary Indication and Procedure	Complication	Time to Complication (mo)
1	65	F	Rotator cuff arthropathy/RSA	AF (Levy II)	1.4
2	55	M	Rotator cuff arthropathy/RSA	AF (Levy II)	4.5
3	52	F	Rotator cuff arthropathy/RSA	SSF (Levy III)	13.5
4	85	M	Proximal humeral fracture/RSA	AF (Levy II)	11.8
5	82	F	End-stage arthrosis/RSA	AF (Levy I)	2.5
6	73	M	Rotator cuff arthropathy/RSA	AF (Levy II)	14.4
7	83	F	Rotator cuff arthropathy/RSA	AF (Levy I)	3.1
8	72	M	Rotator cuff arthropathy/RSA	AF (Levy II)	2.25
9	75	M	Rotator cuff arthropathy/RSA	AF (Levy I)	1.33
10	71	F	Rotator cuff arthropathy/RSA	AF (Levy I)	5.5
Average	71.30	50% M	—	—	6.03

AF/SSF = acromial and scapular spine stress fractures, RSA = reverse total shoulder arthroplasty.

### Patient-Reported Outcome Measures and Active Range of Motion

**Figure 1:**
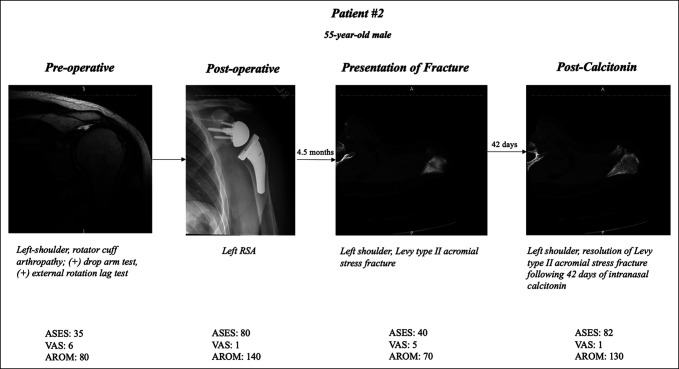
Scans showing the case presentation of patient 2 from preoperative to post-calcitonin treatment. AROM = active range of motion, ASES = American Shoulder and Elbow Surgeons, RSA = reverse total shoulder arthroplasty, VAS = visual analog scale.

Preoperatively, the mean VAS was 7.3, the mean AROM in the forward flexion plane was 90°, and the mean ASES score was 31. Postoperatively, the mean VAS significantly improved to 1 (*P* < 0.001), the mean AROM significantly increased to 128.5° (*P* < 0.001), and the mean ASES score significantly improved to 83.8 (*P* < 0.001). Furthermore, at presentation of fracture, the mean VAS significantly worsened to 7 (*P* < 0.001), the mean AROM significantly decreased to 82° (*P* = 0.009), and the mean ASES score significantly worsened to 37.8 (*P* < 0.001). On completion of the prescribed intranasal calcitonin treatment for AF/SSF, the mean VAS significantly improved to 1.2 (*P* < 0.001), the mean AROM significantly increased to 128° (*P* = 0.008), and the mean ASES significantly improved to 81.4 (*P* < 0.001). The average quantitative change in outcomes from presentation of fracture to completion of intranasal calcitonin treatment was a decrease of 5.8 points in VAS, a gain of 46° in AROM, and a 43.6-point improvement in the ASES score. A summary of PROM and AROM outcomes at each time point, including significant differences, is provided in Table 2. A case presentation of a patient with rotator cuff arthropathy (patient 2) who sustained a Levy type II AF after RSA is illustrated in Figure 1 to delineate the radiographic improvements associated with this treatment modality.

**Table 2:** Summary of Average VAS, ASES, and AROM Outcomes at Each Time Point

Outcome	Preop	Postop	Presentation of Fracture	Post-Calcitonin	Δ Fracture-Calcitonin	*P* Value
VAS	7.3	1	7	1.2	−5.8	<0.001
AROM	90	128.5	82	128	46	0.008
ASES	31	83.8	37.8	81.4	43.6	<0.001

AROM = active range of motion, ASES = American Shoulder and Elbow Surgeons, VAS = visual analog scale.

*P*-values reported are in reference to the statistical significance of the difference between presentation of fracture and post-calcitonin time point data.

*P*-values <0.05 were considered statistically significant.

### Fracture Type and Levy Classification

**Figure 2:**
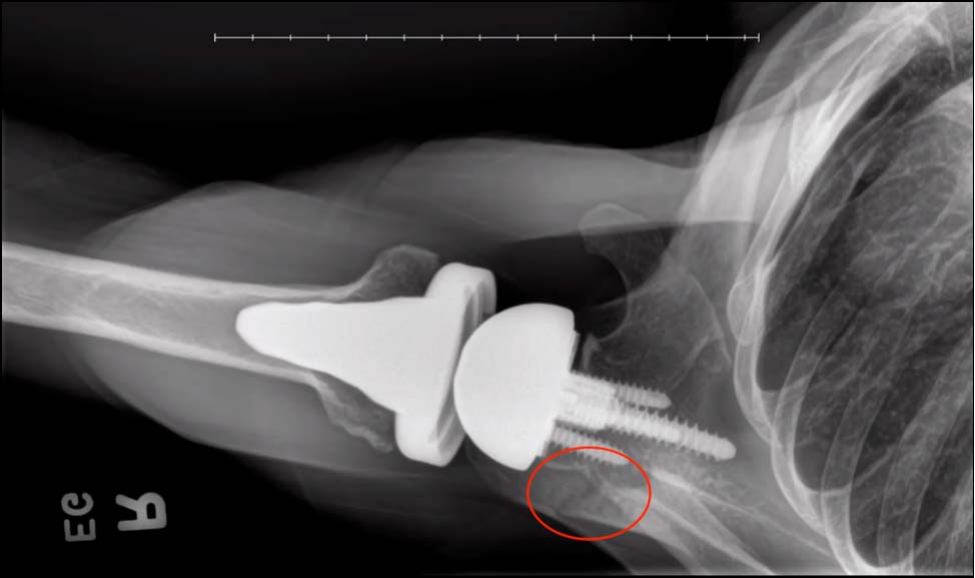
Radiograph of patient 1 at presentation of an acromial fracture, Levy type II.

**Figure 3:**
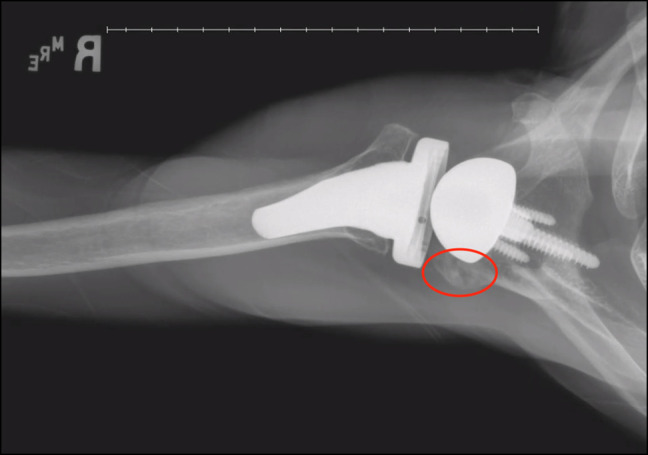
Radiograph of patient 1 after intranasal calcitonin treatment for a Levy II acromial fracture.

Of the 10 total fractures, nine were classified as AFs and one as a SSF. Five of the nine acromial fractures were Levy II, with the remaining four fractures classified as Levy I. The one SSF was classified as Levy III. Figure 2 shows the Levy II AF of patient 1 at presentation of fracture using a radiograph, and Figure 3 shows resolution of this same fracture after treatment with intranasal calcitonin. At the post-calcitonin time point, those with Levy type II/III fractures had significantly higher VAS scores compared with patients with Levy type I fractures (*P* = 0.027). No notable differences were observed between these two groups at the time point for ASES scores and AROM.

## Discussion

Given that the role of RSA has expanded to treat a variety of complex shoulder pathologies, there are numerous complications associated with the surgery.^1,3,8^ Fortunately, the incidence of many of these complications has declined owing to the advancements in prosthesis design, an improved understanding of implant biomechanics, and patient rehabilitation, after RSA.^2,3,14^ However, the incidence of AF/SSFs after RSA has not experienced the same decline as other complications.^3^ Furthermore, it has been shown that the typical treatment course consisting of cessation of physical therapy and immobilization alone are associated with high rates of patient dissatisfaction and worse PROMs (ie, ASES, VAS, single assessment numeric evaluation, simple shoulder test) as compared with control subjects.^5^ Small sample sizes and sparse research may contribute to the lack of consensus on how to manage these fractures nonsurgically.^3,16,18^ Thus, new modalities warrant investigation. Our limited case series demonstrates that after the completion of intranasal calcitonin treatment, the mean VAS, AROM, and ASES scores all markedly improved compared with those at fracture presentation. Although our study did not investigate the efficacy of intranasal calcitonin in comparison with sling immobilization alone, our study reports the early positive results of this novel treatment modality in support of intranasal calcitonin as a viable adjunct nonsurgical modality for nondisplaced AF/SSFs after RSA.

One critical consideration in the nonsurgical management of AF/SSF after RSA is fracture location (Levy classification). Boltuch et al^5^ demonstrated that nonsurgical treatment (sling shoulder immobilization) of patients with medial fracture subtypes (Levy type IIB, IIC, and III) demonstrated markedly worse clinical and radiographic outcomes as compared with control subjects. However, in the nonsurgical treatment of patients with lateral fracture subtypes of AF/SSFs (Levy type I and IIA), patients fared similarly to control subjects for both clinical and radiographic outcomes. To that matter, our patient cohort consisted of nine AFs, of which four were type I, five were type II, and one was type III scapular spine fracture. Moreover, our findings affirm previous literature in that those with fractures medial to the glenoid face (Levy II/III) reported markedly greater VAS pain scores as compared with those with fractures lateral to the glenoid face (Levy I). This is best explained in terms of the uncoupled kinematics that develops in more medial AF/SSFs that involve larger portions of the deltoid, subsequently leading to an upward rotation of the scapula while the acromion translates more inferiorly and ultimately imparts a greater risk of progressive scapular notching and osteolysis at the glenoid neck and lateral scapular pillar. However, given the notable improvement in symptomatic relief provided by the addition of intranasal calcitonin to shoulder immobilization across all fracture subtypes, this treatment modality may help curb worse clinical outcomes associated with just shoulder immobilization alone. Furthermore, Routman et al^26^ showed that those with AF/SSFs treated with sling immobilization alone saw a 34.8-point increase in ASES and a 21.2° increase in active forward flexion, whereas our study demonstrated a 43.6-point increase in ASES and 46° increase in active forward flexion, possibly indicating a synergistic effect of intranasal calcitonin in conjunction to sling immobilization. Thus, this treatment modality may be beneficial to patients with various fracture subtypes, although additional studies with more participants and comparator cohorts are warranted.

Calcitonin has been used to treat osteoporotic fractures, bone mineral disease, hypercalcemia, and Paget disease since gaining US FDA approval in 1995.^27^ Few safety concerns have been raised about the use of calcitonin in its decades of utilization; however, in 2012, the European Medicines Agency suspended the use of intranasal calcitonin, controversially citing calcitonin as having an association with cancer diagnosis.^23,28^ Since its suspension in Europe, several preclinical, preliminary, and clinical studies have investigated the association of calcitonin and cancer—none of which have reported a causal relationship or notable association.^28,29,30,31,32,33,34^ Our results are consistent with the literature, in that routine follow-ups, which consisted of both standard hepatic function tests and blood work, did not reveal any aberrations or signs of cancer at 6-month follow-up.

In addition to its antiresorptive effects, calcitonin may also benefit patients with AF/SSF through its analgesic effects, which have been suggested to occur through an endorphin-mediated mechanism.^35^ Moreover, several studies have demonstrated the efficacy of intranasal calcitonin therapy in treating acute and chronic pain associated with osteoporotic vertebral fractures, as well as preventing their occurrence.^21,36^ Our study did not seek to evaluate rates of bony union or radiographic improvement after treatment with intranasal calcitonin given that the antiresorptive properties of calcitonin have been elucidated; however, we did seek to investigate the role of calcitonin as an analgesic through pain reduction and subsequent improvement in functional status. To that matter, our study demonstrated notable pain reduction and improved functional status after completion of intranasal calcitonin treatment, suggesting an expanded role of this novel off-label treatment modality.

Other reports have described off-label use of various osteoporosis medications for the treatment of AF/SSF after RSA because osteoporosis and bone mineral disease are well-characterized risk factors.^37^ The most widely used antiresorptive drugs for the treatment of osteoporosis are diphosphonates.^20^ While an excellent treatment of bone mineral disease, diphosphonates are associated with several, severe adverse effects, such as osteonecrosis of the jaw, arrhythmias, and severe musculoskeletal pain, and are contraindicated in patients with severe renal dysfunction.^20,38,39^ Other drugs have been proposed as potential adjuvants for both AF/SSF treatment or prevention. Denosumab and teriparatide have been used to treat other types of osteoporotic fractures, and the use of these drugs to treat AF/SSF fractures after RSA have been previously discussed as case reports.^40^ However, limited reports on the use of these drugs in AF/SSF exist, and no clinical studies have been published in the literature.

The results of this study demonstrate that there was a notable improvement in patients' mean VAS scores, AROM, and ASES scores after completing a course of intranasal calcitonin. These findings suggest that administration of calcitonin in patients with these fractures should be considered and may offer additional benefit to patients classically treated with sling immobilization alone. Further application of this study's investigation may provide a useful guide for orthopaedic providers in symptomatic management of patients.

## Limitations

The main limitation of this study is the small sample size. With only 10 patients in this sample, the power of our study's statistics is reduced, and therefore, our results necessitate corroboration through additional investigation. However, the study's main purpose was to report the early results of intranasal calcitonin treatment of AF/SSF after RSA to determine a proof of concept in an effort to incite additional investigation. Moreover, our study chose to focus on patient satisfaction, as measured by pain and functional status, given that current treatment with sling immobilization is associated with patient dissatisfaction as opposed to fracture nonunion.^5^ In addition, this study has limited applicability worldwide. Although FDA approved in the United States, intranasal calcitonin is not available in Europe per the European Medicines Agency, and therefore, the results of this study are specific to US patients. To that matter, we did not seek to investigate the full safety profile of intranasal calcitonin and instead followed patients for any adverse medical complications by standard blood work and hepatic function testing. Moreover, this is a retrospective study of completed treatment based on surgeon preference. This study only followed patients who were diagnosed with AF/SSF and subsequently treated with intranasal calcitonin based on surgeon preference; patients who received RSA later in our inclusion time line may develop AF/SSF; however, these patients were not included because their complication would occur outside of our study's time line.^41,42^ Additional prospective studies with a control/comparative group, larger patient cohorts, and longer follow-up are necessary to comment on the clinical significance of our findings. Instead, this study relies on existing literature to make qualitative comparisons of the intranasal calcitonin treatment modality with other nonsurgical treatments.

## Conclusions

The use of intranasal calcitonin was not associated with adverse events including no aberrations/signs of cancer at 6-month follow-up after administration. Calcitonin with sling immobilization markedly improved clinical and functional outcomes of patients with nondisplaced AF/SSF and may be considered by orthopaedic surgeons for symptom management.
